# Aging modifies receptor expression but not muscular contractile response to angiotensin II in rat jejunum

**DOI:** 10.1007/s13105-022-00892-7

**Published:** 2022-04-08

**Authors:** Maria Grazia Zizzo, Adele Cicio, Federica Corrao, Laura Lentini, Rosa Serio

**Affiliations:** 1 Department of Biological, Chemical and Pharmaceutical Sciences and Technologies (STEBICEF), University of Palermo, Viale delle Scienze, ed. 16, 90128 Palermo, Italy; 2 ATeN (Advanced Technologies Network) Center, University of Palermo, Viale delle Scienze, ed.18, 90128 Palermo, Italy

**Keywords:** Angiotensin II, Angiotensin II receptors, Intestinal motility, Aging

## Abstract

The involvement of renin-angiotensin system in the modulation of gut motility and age-related changes in mRNA expression of angiotensin (Ang II) receptors (ATR) are well accepted. We aimed to characterize, in vitro, the contractile responses induced by Ang II, in jejunum from young (3–6 weeks old) and old rats (≥ 1 year old), to evaluate possible functional differences associated to changes in receptor expression. Mechanical responses to Ang II were examined in vitro as changes in isometric tension. ATR expression was assessed by qRT-PCR. Ang II induced a contractile effect, antagonized by losartan, AT1R antagonist, and increased by PD123319, AT2R antagonist, as well by neural blocker ω-conotoxin and by nitric oxide (NO) synthase inhibitor. No difference in the response was observed between young and old groups. AT1 receptor-mediated contractile response was decreased by U-73122, phospholipase C (PLC) inhibitor; or 2-aminoethoxy-diphenylborate (2-APB), inositol triphosphate (IP_3_) receptor inhibitor; or nifedipine, l-type calcium channel blocker. Age-related changes in the expression of both AT1 receptor subtypes, AT1a and AT1b, and of AT2 receptors were detected. In conclusion, Ang II modulates the spontaneous contractility of rat jejunum via postjunctional AT1 receptors, involving Ca^2+^ mobilization from intracellular stores, via PLC/IP_3_ pathway, and Ca^2+^ influx from extracellular space, via l-type channels. Prejunctional AT2 receptors would counteract AT1 receptor effects, via NO synthesis. The observed age-related differences in the expression of all AT receptor subtypes are not reflected in the muscular contractile response to Ang II.

## Introduction

The renin-angiotensin system (RAS), with its key mediator angiotensin II (Ang II), is well-known as an endocrine system, mainly involved in the regulation of systemic blood pressure. However, RAS components can play local, tissue-level functions, as local blood perfusion regulation, tissue remodeling and wound healing, extracellular fluid volume, and electrolyte homeostasis [8].The presence of local RAS in different parts of the gastrointestinal (GI) tract has been demonstrated and its function and regulation are becoming clearer [11]. Locally produced angiotensin II, acting as paracrine regulator, influences the mucosal net fluid and buffer transport [13–17] as well as regulates the GI muscular wall contractility [21, 22, 25]. The effects of Ang II are mediated by specific G-coupled membrane receptors classified as AT1 and AT2 receptors. In rodents at least two distinct genes (AT1a and AT1b) encoding type 1 angiotensin II (AT1) receptors were identified. AT1 and AT2 receptor subclasses show difference in the relative binding to Ang II and to Ang II proteolytic fragments and in the coupled intracellular biochemical signaling pathway.

The signal transduction mechanisms coupled to AT1 receptors relay on at least five different effectors: phospholipase C (PLC), voltage-dependent Ca^2+^ channels, phospholipase D, phospholipase A2, and adenylate cyclase. It has also been demonstrated that AT1R stimulates the phosphorylation of several tyrosine-containing proteins, such as mitogen-activated protein (MAP) kinase, and activates the JAK-STAT pathway [7]. The coupling between AT2 receptors and the intracellular signaling apparatus remains unclear, although an interaction with nitrergic pathway has been reported [1]. Accumulating evidence suggests that AT2 receptor in general not only opposes the AT1 receptor-induced response, but also it has its own effects independent of interaction with AT1 receptor signaling. Several studies indicate a change in the expression and/or in the involvement of AT2 receptors during aging, with a reduction at the transition from fetal to newborn life. AT1 receptors, on the contrary, are expressed later in development and persisted throughout adult life [2, 6, 28].

In the GI tract, RAS expression has been previously reported in rodent intestine [17, 21, 25]; moreover, Pasanen et al. [24] in a recent study characterize in the intestinal RAS expression in young and adult rats, reporting that jejunal AT1R expression was increased in age-related manner. Since AT1 receptors are mainly involved in intestinal motor effects of Ang II in the gut of different species including rodents, we aimed to characterize the response to Ang II in the jejunum of young and old rats, hypothesizing possible differences in the contractile response. We also investigated the action mechanism underlying the Ang II effects, emphasizing the role of calcium movements triggered by AT1 receptor stimulation. Finally, we evaluated using qRT-PCR, the possible age-dependent changes of the expression of both AT1a and AT1b receptor subtypes and of AT2 receptors.

## Materials and methods

### Effects of Ang II on isolated rat jejunum

All animal procedures and care were approved by the Animal Care and Use Ethics Committee of the University of Palermo (authorization number: 69636.NJCO–NTRW) and performed in accordance with national and EU guidelines for the handling and use of experimental animals.

In vitro mechanical activity of isolated intestinal segments was measured using the isolated tissue bath technique as previously described [29]. Briefly, young (3–6 weeks old, 120–150 g) and adult Wistar rats (≥ 1 year old, 250–300 g) (ENVIGO Srl, San Pietro al Natisone UD, Italy) were euthanized using 2% isoflurane anesthesia followed by cervical dislocation. After laparotomy, jejunum was rapidly excised and placed in Krebs solution. Full-thickness segments from jejunum (about 15 mm in length) were isolated and suspended; longitudinally oriented, in 10 mL four-channel organ bath containing oxygenated (95% O_2_ and 5% CO_2_) and warmed (37 °C) Krebs solution; and anchored at the distal end to an organ holder and at the proximal end secured with a silk thread to a force transducer (FORT 10, Ugo Basile, Biological Research Apparatus, Comerio VA, Italy) for isometric recording of muscular activity (PowerLab/400 system, Ugo Basile, Italy). Preparations, subjected to an initial tension of 1 g, were allowed to equilibrate for at least 40 min to develop rhythmic spontaneous contractions.

After the equilibration period, preparations were challenged with 0.1 µM isoprotenerol, adrenergic receptor agonist, or with 10 µM carbachol (CCh), muscarinic receptor agonist, until stable relaxant or contractile responses were obtained.

Concentration-response curves for Ang II (0.1–300 nM) were constructed by non-cumulative addition. Ang II was applied for approximately 5-min at 40-min intervals to avoid desensitization of the receptors. Time control experiments showed that a second curve to the agonist was reproducible.

The concentration-response curve to Ang II (0.1–300 nM) was repeated in the presence of AT receptor antagonists losartan (100 nM) (AT1 receptor antagonist) or PD123319 (100 nM) (AT2 receptor antagonist) leaved in contact with the tissue for at least 30 min before testing Ang II.

In a second set of experiments, the effects of ω-conotoxin (100 nM) (Ca^2+^ voltage-gated neural channel blocker), atropine (1 μM) (cholinergic muscarinic receptor antagonist), or l-NAME (100 μM) (a blocker of the NO synthase) were tested against a submaximal dose of Ang II (50 nM). Lastly, to characterize the source of the Ca^2+^ underlying the contractile effects, the response to the submaximal dose of Ang II was tested in the presence of nifedipine (5 nM) (l-type Ca^2+^-channel blocker), 2-APB (20 μM) (IP_3_ receptor blocker), U-73122 (10 μM) (PLC inhibitor), or ryanodine (10 μM) (ryanodine-sensitive intracellular calcium store blocker). Each preparation was tested with a single antagonist or blocker, except when otherwise stated. Concentrations of the drugs used were determined from preliminary experiments and from the literature. All the drugs were leaved in contact with the tissue for at least 30 min before testing Ang II.

The composition of Krebs solution was (mM) NaCl 119; KCl 4.5; MgSO4 2.5; NaHCO_3_ 25; KH_2_PO_4_ 1.2; CaCl_2_ 2.5; and glucose 11.1. The following drugs were used: atropine sulfate; 2-aminoethoxy-diphenylborate (2-APB); Carbamoylcholine chloride (carbachol); nifedipine; N(ω)-nitro-L-arginine methyl ester (L-NAME); ryanodine; 1-[6((17β-3-methoxyestra-1,3,5(10)-trien-17-yl)amino)hexyl]-1H-pyrrole-2,5-dione} (U-73122) ω-conotoxin (Sigma-Aldrich, Inc., St. Louis, MO, USA); angiotensin II; 1-[[4-(dimethylamino)-3-methylphenyl]methyl]-5-(diphenylacetyl)-4,5,6,7-tetrahydro-1H-imidazo[4,5-c] pyridine-6-carboxylic acid ditrifluoroacetate (PD123319); and 2-butyl-4-chloro-1-[[2′-(1Htetrazol-5-yl)-[1,1′-biphenyl]-4-yl]methyl]-1H-imidazole-5-methanol potassium salt (losartan) from Tocris Bioscience (Bristol, UK). All the drugs were dissolved in distilled water, except for nifedipine and 2-APB which were dissolved in ethanol and ryanodine and U-73122 which were dissolved in dimethyl sulfoxide. The working solutions were prepared fresh on the day of the experiment by diluting the stock solutions in Krebs. Control experiments using the different solvents alone showed that none had effects on the spontaneous contractile activity.

### RNA preparation and real-time PCR analysis

Total RNA was extracted from young and old whole jejunum using RNeasy Mini kit according to manufacturer’s instruction (Qiagen, Milano, Italy). RNA was reverse-transcribed in a final volume of 50 μL using the High-Capacity cDNA Archive kit (Applied Biosystems, Monza, Italy) for 10 min at 25 °C and 2 h at 37 °C. For quantitative SYBR Green real-time PCR, the reaction was carried out in a total volume of 25 μL containing 2×SYBR Green I Master Mix (Applied Biosystems), 2 μL of cDNA, and 300 nM forward and reverse primers [20] using the ABI PRISM 7300 instrument (Applied Biosystems, Monza, Italy). The oligonucleotides used are reported in Table 1.

**Table 1:** Sequences of primers used for qRT-PCR [20]

Gene description	Forward primer sequence	Reverse primer sequence
β-Actin	CTAAGGCCAACCGTGAAAAG	GCCTGGATGGCTACGTACA
AT1a	CCATTCACCCTGCCTCAG	ACGGCTTTGCTTGGTTACTC
AT1b	ATGTCTCCAGTCCCCTCTCA	TGACCTCCCATCTCCTTTTG
AT2R	CAATCTGGCTGTGGCTGACTT	TGCACATCACAGGTCCAAAGA

Relative changes in the target mRNA between young and old samples were determined using the ∆∆Ct method. Levels of the target transcripts were normalized to β-actin, a housekeeping gene constantly expressed in all samples (∆Ct). Final values were expressed as 2^^−(ΔCt)^ versus β-actin

### Statistical analysis

All data are given as means ± S.E.M.: “*n*” in the “Results” section refers to the number of animals on which observations were made. The excitatory responses induced by Ang II were estimated as increase in tension above the basal tone set as baseline and reported as a percentage of the increase of tone induced by 10 μM CCh. Contractile responses were fitted to sigmoid curves (Prism 5.0, Graph-PAD), and EC_50_ values with 95% confidence limits (CLs) were determined. Statistically significant differences were calculated by Student’s *t* test or by means of analysis of variance, followed by Bonferroni’s test, when appropriate. A probability value of 0.05 was regarded as significant.

## Results

### Effects of Ang II on the spontaneous mechanical activity

Segments of rat jejunum, once mounted in the organ bath, developed a spontaneous activity characterized by rhythmic contractions with amplitude significantly higher in the old compared to young group (481.3 ± 49.7 mg (*n* = 9) in the young group vs 761.7 ± 53.1 mg in the old group (*n* = 9), *P* < 0.05). No difference has been found in the frequency of the contraction between the groups, being 25.3 ± 0.1 cpm (contractions per minute) in the young group (*n* = 9) and 26.2 ± 0.5 cpm in the old group (*n* = 9).

**Fig. 1:**
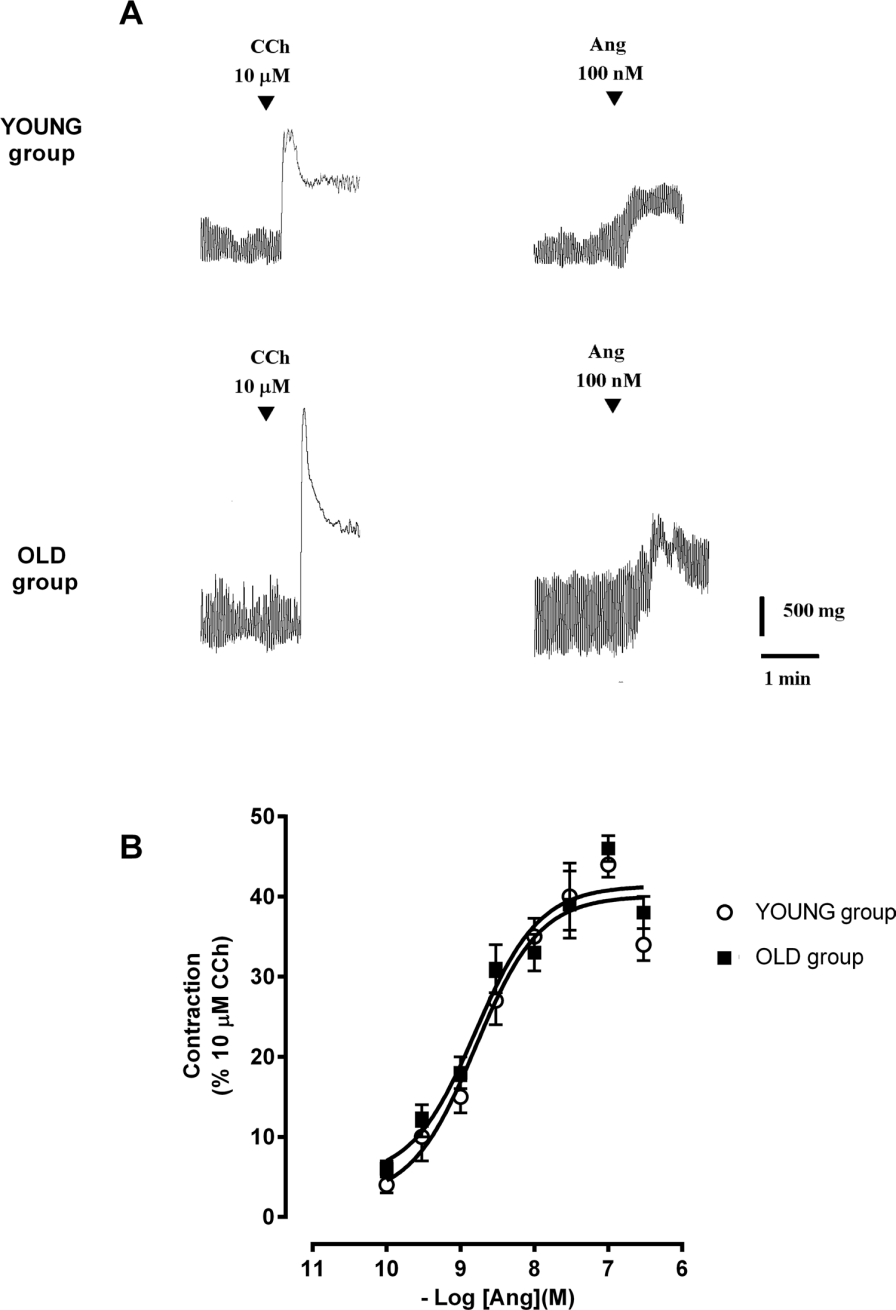
Ang II responses in young and old jejunum preparations. **A** Original recordings showing the mechanical responses evoked by 10 μM CCh and 100 nM Ang II in jejunum of young and old rats. **B** Concentration-response curve for the excitatory effects induced by Ang II (0.1–300 nM) in jejunum of young and old rats. Data are means ± S.E.M. and are expressed as percentage of the maximal effect induced by 10 μM CCh (*n* = 9 for each group)

The contractile response induced by 10 µM CCh was also higher in the old group compared to the young group (2.47 ± 0.65 g vs 1.70 ± 0.43 g, respectively (*n* = 9 each), *P* < 0.05). Ang II (0.1–300 nM) caused a concentration-dependent contractile effect in the jejunal segments from both groups of animals. The maximal response to Ang II was observed at the dose of 100 nM (absolute increase in muscular tone was 0.88 ± 0.26 g in young vs 1.3 ± 0.63 g in old animals; *n* = 9 each). No effect on the frequency was observed. The contractile effect persisted throughout the application of the drug and was reversible after washout (Fig. 1A). When normalized to the contractile response to 10 μM CCh, no difference in the potency or in the efficacy of Ang II was observed between the groups (Table 2, Fig. 1B).

**Table 2:** Effects of Ang II before and after treatments with antagonists

	Young group				Old group			
	EC_50_	95% Cl	EMAX (% 10 μM CCh)	*n*	EC_50_	95% Cl	EMAX (%10 μM CCh)	*n*
Ang II	6.7 nM	3.1–20 nM	45.5 ± 2.8	9	3.9 nM	1.3–10.4 nM	46.0 ± 3.4	9
Ang II + losartan	31.4* nM	13–71 nM	39.2 ± 4.6	5	21.5* nM	9.5–48.2 nM	40.1 ± 4.2	5
Ang II + PD123319	8.8 nM	2.5–14.4 nM	64.7 ± 3.2*	5	2.9 nM	1.5–5.4 nM	61.8 ± 4.3*	5

^*^P < 0.05 compared to the respective own control condition

**Fig. 2:**
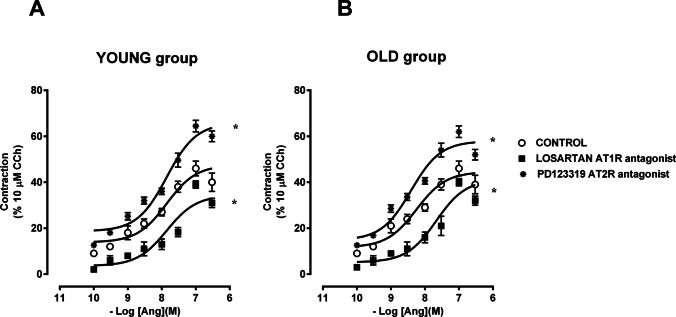
Characterization of receptors involved in excitatory effects induced by Ang II in young and old jejunum preparations. Concentration-response curves to Ang II (0.1–300 nM) before and after losartan, AT1 receptor antagonist (100 nM, *n* = 5 for each group), and PD123319 (100 nM, *n* = 5 for each group), AT2 receptor antagonist, in jejunum from young (**A**) and old (**B**) rats. Data are means ± S.E.M. and are expressed as percentage of the maximal effect induced by 10 μM CCh. The values for the control curves are the means of the control data obtained before each treatment. *P < 0.05 compared to the respective own control condition

The contractile response to Ang II in the preparations from both groups was significantly antagonized by losartan (100 nM), AT1 receptor antagonist, which per se did not modify the amplitude nor the frequency of spontaneous activity, shifting the concentration-response curve of Ang II to the right (Table 2; Fig. 2). On the contrary, PD123319 (100 nM), AT2 receptor antagonist, in both preparations induced an increase of the response to Ang II of about 40 % without significantly affecting the amplitude or the frequency of the spontaneous mechanical activity (Table 2; Fig. 2).

**Fig. 3:**
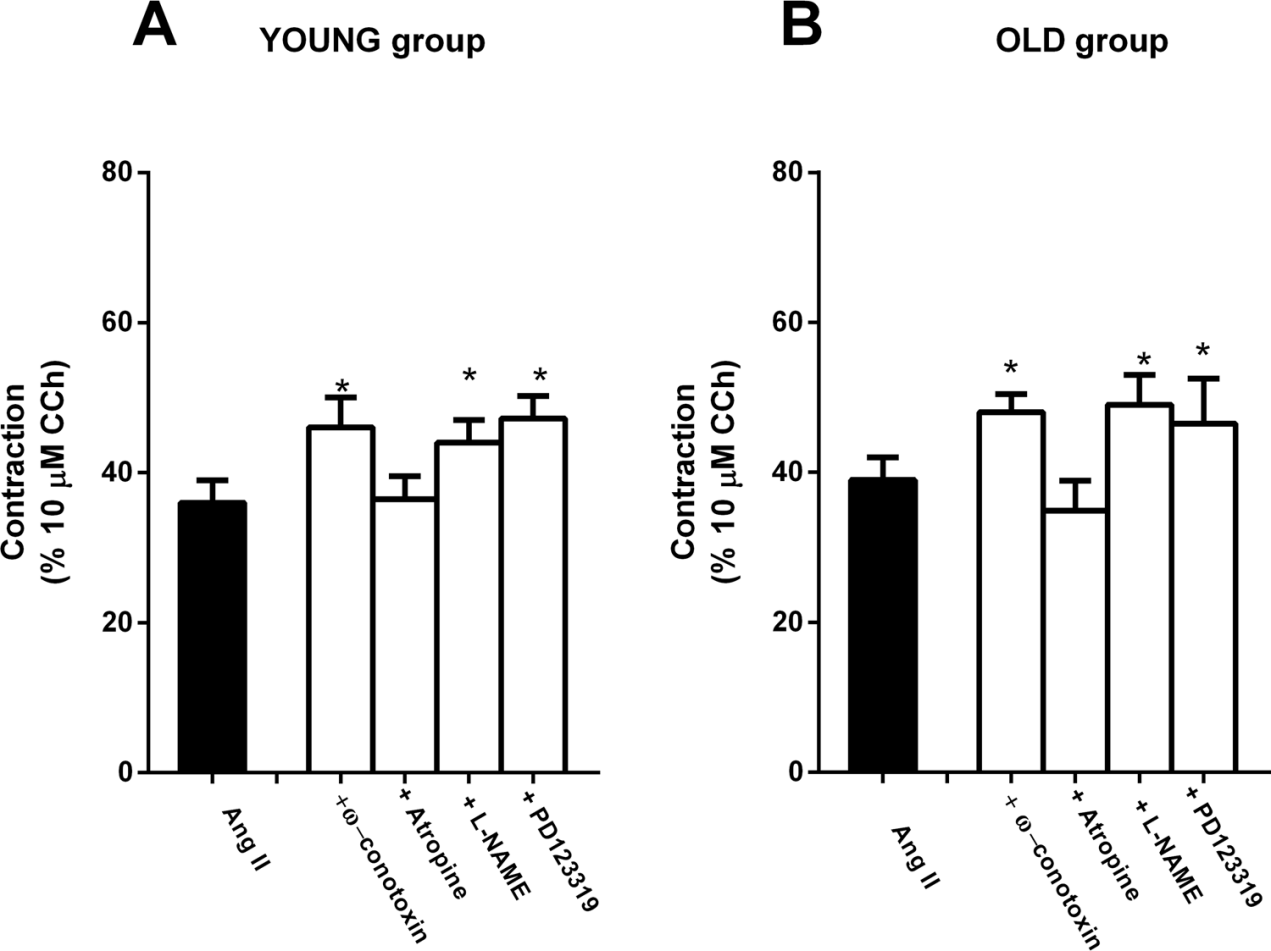
Effects of inhibitor of enteric nervous system pathways and AT2 receptor antagonist on Ang II response. Histogram showing the effects of Ang II (50 nM) in the presence or absence of ω-conotoxin, Ca_2+_ voltage-gated neural channel blocker (0.1 μM, *n* = 4 for each group); or atropine (1 μM, *n* = 4 for each group), muscarinic receptor antagonist; or l-NAME (100 μM, *n* = 4 for each group), inhibitor of NO synthase; or PD123319 (100 nM, *n* = 5 for each group) in jejunum from young (**A**) or old (**B**) rats. Data are means ± S.E.M. and are expressed as percentage of the maximal effect induced by 10 μM CCh. The graphed value for the control bar is the mean of the control data obtained before each treatment. *P < 0.05 compared to the respective own control condition

In the preparations of both groups, the contractile response to Ang II (50 nM) was significantly increased also in the presence of ω-conotoxin (0.1 μM) (Fig. 3) or l-NAME (100 μM) (Fig. 3). No one of the tested blockers affected amplitude nor frequency of the spontaneous activity.

The increase in the response observed in the presence of PD123319 was comparable to that induced by ω-conotoxin or by l-NAME (Fig. 3).

Atropine (1 μM), which per se reduced the spontaneous contractile activity, was ineffective on Ang II–evoked contractile response (Fig. 3).

### Signaling pathway and calcium movements triggered by stimulation of AT1 receptors

**Fig. 4:**
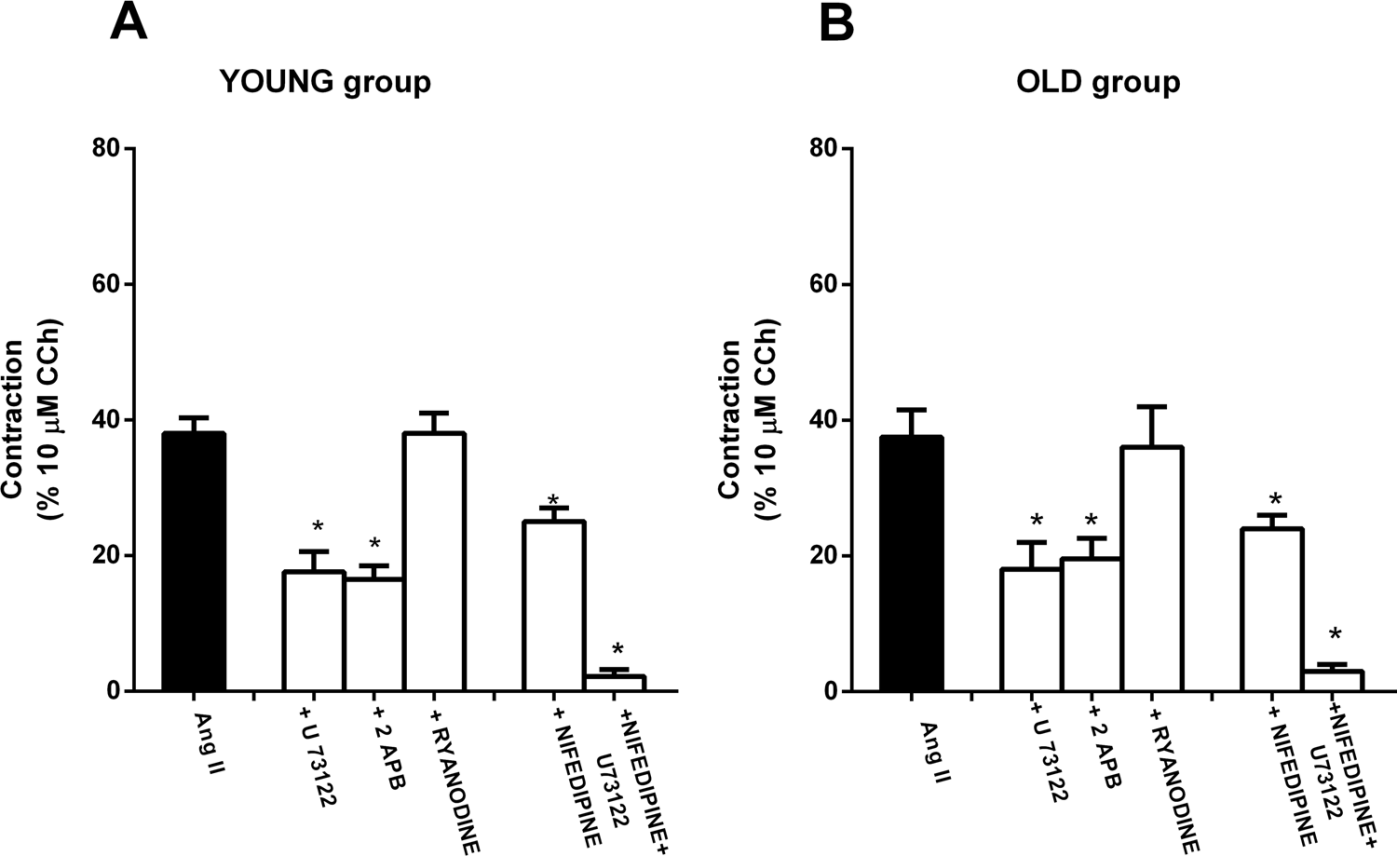
Intracellular signaling involved in Ang II response. Histogram showing the effects of Ang II (50 nM) in the presence or absence of U-73122, PLC inhibitor (10 μM, *n* = 4 for each group); 2-APB, membrane-permeant IP3 receptor inhibitor (20 μM, *n* = 4 for each group); ryanodine (10 μM, *n* = 4); and nifedipine, voltage-gated calcium channel antagonist (5 nM, *n* = 4 for each group), in jejunum from young (**A**) or old (**B**) rats. Data are means ± S.E.M. and are expressed as percentage of the maximal effect induced by 10 μM CCh. The graphed value for the control bar is the mean of the control data obtained before each treatment. *P < 0.05 compared to the respective own control condition

In the preparations from both groups, the contractile effect of submaximal concentration of Ang II (50 nM) was significantly attenuated in the presence of U-73122 (10 μM), as well as after pretreatment with 2-APB (20 μM) (Fig 4).

To exclude possible aspecific effects, 2-APB was tested against CCh, which increases IP_3_ synthesis and release of Ca^2+^ from IP_3_-sensitive Ca^2+^ stores. Contractile effects of CCh (10 μM) were significantly attenuated in the presence of 20 μM 2-APB (data not shown).

Subsequently, to evaluate the possible contribution of extracellular calcium, the effects of the voltage-gated calcium channel antagonist, nifedipine, on Ang II–induced contraction were investigated. Nifedipine (5 nM), at such relatively low concentrations, did not affect the spontaneous activity, but reduced the response to Ang II by 32 and 34 % of the control values, in young and old groups, respectively. Nifedipine concentrations higher than 5 nM suppressed the spontaneous mechanical activity of the preparations and thereby they could not be tested.

The joint application of 5 nM nifedipine and 10 μM U-73122 abolished the response to Ang II (Fig 4) without significantly affecting the amplitude or the frequency of the spontaneous mechanical activity.

Finally, in our preparations, pretreatment with ryanodine (10 μM) did not affect the response of Ang II (Fig 4).

### AT receptor mRNA expression in young and adult rats

As already reported, Pasanen et al. [24] demonstrated that jejunal AT1 receptor expression increased in adult rats as compared to young rats. Since in rodents at least two distinct genes (AT1a and AT1b) encoding AT1 receptors have been identified, we evaluated using qPCR the possible age-dependent changes of the expression of both AT1a and AT1b receptor subtypes. Moreover, a possible age-dependent difference in the expression of AT2 receptors was also explored.

**Fig. 5:**
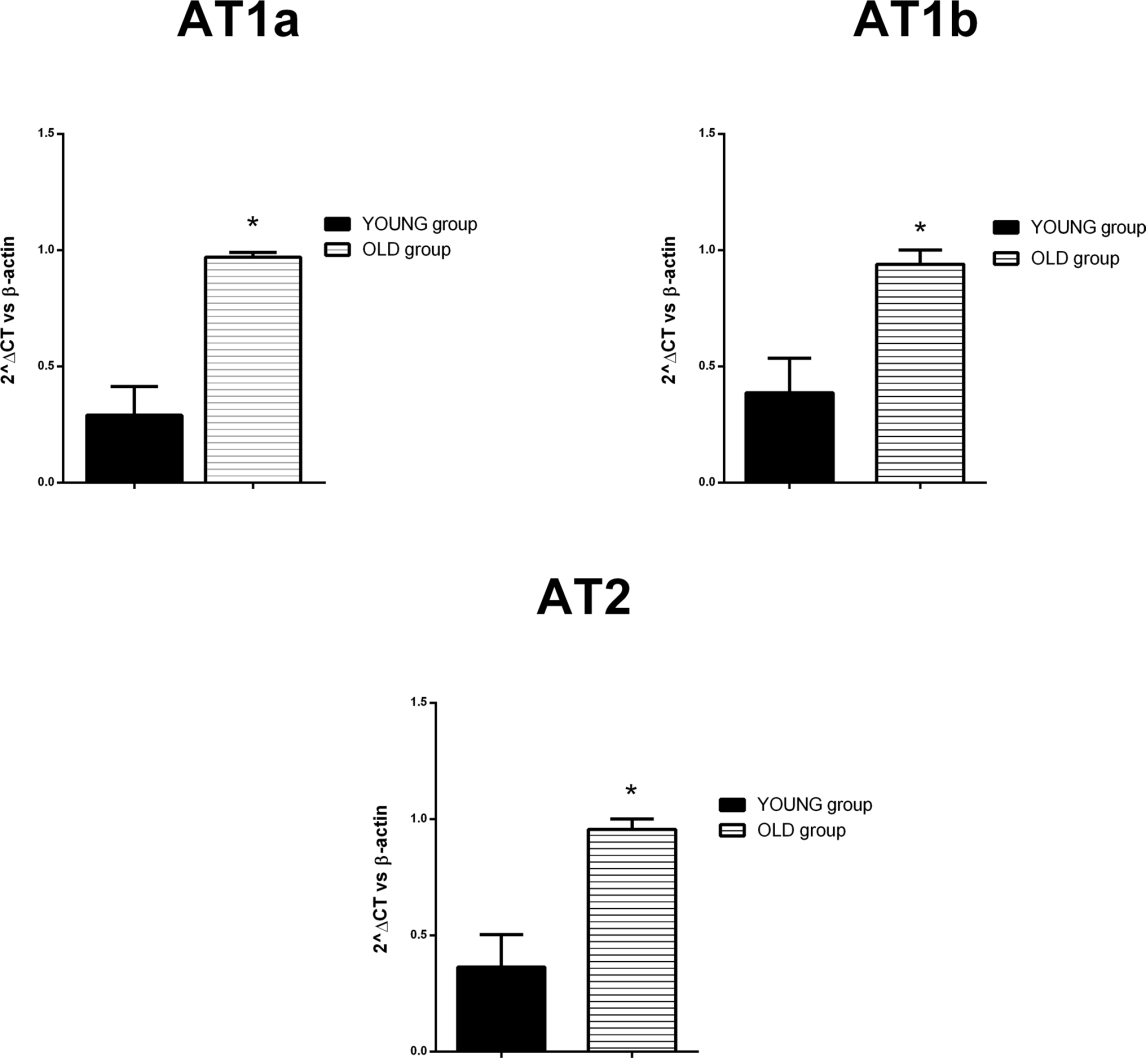
Angiotensin II receptor expression analysis. Histograms showing levels of AT1a (**A**), AT1b R (**B**), and AT2 (**C**) mRNA expression in jejunum from young or old rats. Gene expression was normalized by β-actin. Results are expressed as mean ± S.E.M., *n* = 3 each. *P < 0.05 compared to old group

mRNA expression of the investigated AT receptors was present in the jejunum at all ages. In the old group, there is an increase in the mRNA expression of all the AT receptor subtypes investigated (Fig 5).

## Discussion

Results from the present study indicate that in rat jejunum, there is an age-related change in the expression of both AT1 receptor subtypes, AT1a and AT1b, and of AT2 receptors. However, these differences are not reflected in the muscular contractile response to exogenously administered Ang II. Ang II, activating postjunctional AT1 receptors, is able to contract jejunum smooth muscle through multiple steps, involving Ca^2+^ mobilization from intracellular stores, via PLC/IP_3_ pathway, and Ca^2+^ influx from extracellular space, via l-type calcium channels. Concurrently, activation of AT2 receptors would counteract the excitatory effects mediated by AT1 receptor, via enteric nerves and increased NO production.

Age-dependent changes in the expression of the various components of RAS have been observed in lungs, skeleton, and vasculature and associated with the age-dependent degeneration in the kidney and cardiovascular system [4, 27]. A correlation between AT1 receptor expression and detrimental effects in aging was suggested by Benigni [5], who reported that targeted disruption of the Agtr1a gene, that encodes AT1a receptors, promotes longevity in mice. Previously, few studies reported changes in RAS expression with age also in the gut showing an age-related increase in ACE1-AT1R axis [12, 24]. Our results not only confirm data about the age-related changes in the AT1 receptor mRNA expression, but also indicate that both the AT1 receptor subtypes (AT1a and AT1b) undergo changes in the expression with age. Moreover, age-related difference in AT2 receptor expression has been also observed. Therefore, we aimed to characterize the contractile response to Ang II in the jejunum from young and old animals and to point out eventual difference in the response with the age.

As already observed in different gut preparations from human and animal models [9, 21, 22, 25, 29], our data demonstrated that, also in the rat jejunum, Ang II is involved in the modulation of the smooth muscle contractility. In particular, Ang II induced a consistent dose-dependent contractile response regardless of whether the jejunum was from young or old rats. Indeed, the absolute contraction evoked by Ang II was higher in the old group. Such an observation could be ascribed to the increase expression of AT receptors. However, when normalized to the maximal response of CCh obtained in the same tissue, the response to Ang II did not differ in terms of potency or efficacy between the two groups. Therefore, we feel that a widespread modification of the contractile apparatus is responsible for the enhancement of both spontaneous and pharmacologically evoked contractile activity in the longitudinally oriented jejunum segments from old groups.

The contractile effect is mediated by AT1 receptors in both preparations, as suggested by the significant reduction of the response observed in the presence of losartan, AT1 receptor antagonist. Interestingly, in both preparations, the contractile response to Ang II was significantly increased by pretreatment with PD123319, AT2 receptor antagonist, confirming the notion that AT2 receptor activation would counteract the excitatory effects mediated by AT1 receptors [23]. Indeed, counteracting effects of AT1 and AT2 receptor activation have been reported in rat ileum [23]. A recruitment of Ang II AT2 receptors counteracting AT1R-contractile activity was evident in inflamed tissue in longitudinal strips of distal rat colon [29] and AT2 receptor involvement was suggested in whole rat colon [10]. Moreover, the inhibitory effects induced by AT2 receptors have been attributed to the release of inhibitory autacoids such as NO [1, 26]. Data from the present experiments strengthen the hypothesis of the involvement of NO in the inhibitory effects due to activation of AT2 receptors since the inhibition of NO synthesis by l-NAME induced an increase of Ang II–mediated contraction at a level similar as in the presence of PD123319. A similar increase in the contractile response to Ang II was observed in the presence of the Ca^2+^ voltage-gated neural channel blocker, ω-conotoxin, suggesting that AT2 receptors might be located on enteric neurons leading to the synthesis of NO, thereby reducing the excitatory effects due to AT1 receptor activation. AT2-mediated inhibitory effects via nitrergic neural pathway have been also reported in rat anococcygeus smooth muscle and in inflamed colon [8, 29].

Ang II–induced contractile response remains in the presence of ω-conotoxin or in the presence of atropine indicating that neither enteric nerves nor ACh is involved in the AT1 receptor mediating excitatory effects. Thereafter, in the preparations from both groups, a likely postjunctional localization of AT1 receptors can be suggested.

Actually, we expected that changes in mRNA expression of AT receptors could reflect a difference in the response to Ang II in the preparations from young and old groups. Indeed, no age-related changes were observed in the features of contractile responses, nor in the potency or efficacy of Ang II, nor in the antagonism of Ang II receptors. However, our results and Pasanen and collaborator studies [24] have been conducted on whole-thickness muscular wall, thereby including all intestinal layers, neurons, and blood vessels. Therefore, the increase in the expression of AT receptors may be confined to layers different of musculature, as mucosa layer where Ang II is able to stimulate sodium and water absorption [17]. Moreover, increase in gene expression may be not related to an increase of functional protein levels. It is possible also to speculate that the production of higher levels of AT receptor functional proteins could be induced in age-related pathological conditions as inflammation. Further studies are needed to solve this issue.

The second part of this study was performed to investigate the mechanism underlying contractile effects induced by Ang II, and, since it is reported that the aging can affect calcium homeostasis [3, 18], eventual possible differences in calcium mobilization between young and old preparations were analyzed.

Among the different signal transduction mechanisms, binding of AT1 receptors by Ang II can result in the activation PLC/IP_3_ pathway, thereby leading to increase in intracellular Ca^2+^ and muscular contraction. Moreover, different Ca^2+^ entry channels, such as voltage-operated channels and store-operated channels, can participate to the elevation of intracellular Ca^2+^ concentration. In rat jejunum preparations, from both age groups, pretreatment with PLC blocker or IP_3_ receptor antagonist significantly reduced the Ang II contractile response, indicating that, as already observed in different gut preparations [19], downstream mechanism of AT1 receptor activation includes phospholipase C and elevation of Ca^2+^ cytoplasmatic released from IP3 intracellular store.

In addition, in both young and old jejunum, the Ang II–induced contraction was partially reduced by nifedipine, l-type calcium channel blocker, also suggesting an involvement of Ca^2+^ influx from extracellular spaces. Noteworthy, the joint application of U-73122 and nifedipine abolished the response to Ang II.

Moreover, in our preparations, the possibility that Ca^2+^ influx from extracellular space could trigger a Ca^2+^-induced Ca^2+^ release (CICR) from ryanodine-sensitive stores, thereby contributing to contractile response, can be discarded since pretreatment with ryanodine did not affect the response of Ang II.

Although the response of aged tissue to contractile agents, CCh and Ang II, is higher than young tissue, implying alterations in calcium homeostasis with the age as reported by Lopes et al. [18], we did not observe any differences in Ang II action mechanisms with no changes in sensitivity to intracellular calcium store blockers.

In conclusion, Ang II modulates contractile activity of the rat jejunum smooth muscle both in young and old animals via postjunctional AT1 receptor. Activation of AT1 receptor is multiphasic, involving Ca^2+^ mobilization from intracellular stores via PLC/IP_3_ pathway and Ca^2+^ influx from extracellular spaces via l-type calcium channels. Ang II enrolls also AT2 receptors which, via enteric nerves and production of NO, counteract AT1 receptor excitatory effects. Although changes in age-related manner in the expression of all AT receptor subtypes have been observed, these differences are not reflected in the muscular contractile response to exogenously administered Ang II. We feel that our results may serve as basis for further studies to understand the role of AT receptors in the modification of intestinal functions with age.
